# The Effects of Vitamin E and Omega-3 PUFAs on Endothelial Function among Adolescents with Metabolic Syndrome

**DOI:** 10.1155/2014/906019

**Published:** 2014-07-20

**Authors:** Alireza Ahmadi, Mojgan Gharipour, Gholamreza Arabzadeh, Payam Moin, Mahin Hashemipour, Roya Kelishadi

**Affiliations:** ^1^Preventive Pediatric Cardiology Department, Isfahan Cardiovascular Research Center, Isfahan Cardiovascular Research Institute, Isfahan University of Medical Sciences, Isfahan, Iran; ^2^Metabolic Syndrome Department, Isfahan Cardiovascular Research Center, Isfahan Cardiovascular Research Institute, Isfahan University of Medical Sciences, Isfahan, Iran; ^3^Isfahan University of Medical Sciences, Isfahan, Iran; ^4^Endocrine Research Center, Isfahan University of Medical Sciences, Isfahan, Iran

## Abstract

*Aim*. The present study aims to explore the effects of vitamin E and omega-3 on endothelial function indicators among adolescents with metabolic syndrome.* Method*. In a randomized, double blind, and placebo-controlled trial, 90 young individuals, aged 10 to 18 years, with metabolic syndrome were randomly assigned to receive either vitamin E tablets (400 IU/day) or omega-3 tablets (2.4 gr/day) or placebo. For assessing endothelial functional state, the serum level of vascular endothelial growth factor (VEGF) was measured by ELISA test.* Results*. The use of omega-3 supplementation for eight weeks led to significant increase in serum HDL level compared with the group treated with vitamin E or placebo group. In this regard, no significant correlations were found between the change in VEGF and baseline levels of other markers including anthropometric indices and serum lipids. Omega-3 could significantly reduce VEGF with the presence of other baseline variables (Beta = −12.55; *P* = 0.012).* Conclusion*. The administration of omega-3 can effectively improve endothelial function in adolescents with metabolic syndrome by reducing the level of serum VEGF, as a major index for atherosclerosis progression and endothelial destabilization. Omega-3 can be proposed as a VEGF antagonist for improving endothelial function in metabolic syndrome. The clinical implications of our findings should be assessed in future studies.

## 1. Introduction

Arteriosclerosis is initiated by inflammatory processes in the endothelial cells of the vessel wall and leads to metabolic syndrome [1]. Early markers of atherogenesis such as endothelial dysfunction have been associated with the presence of the metabolic syndrome in adults, and a small number of reports show comparable findings in children manifesting the syndrome [2]. Endothelium of major vessels plays essential roles in different vital pathways including hemostasis, regulation of vascular-cellular transportation of metabolites, angiogenesis, and controlling vascular tone [3]. Hence, any impairment in vascular endothelial cells results in arterial stiffness and remodeling leading development of cardiovascular disorders [4]. Endothelial dysfunction is frequently defined as reduction of the bioavailability of vasodilator biomarkers such as nitric oxide along with the increase of the mediators of vasoconstriction [5]. The certain basis of endothelial dysfunction has not been exactly demonstrated; however, the association between derangements and deregulation of metabolic pathways in this condition has been now considered. In this context, a variety of metabolic disturbances have been identified to contribute to endothelial dysfunction such as hyperinsulinemia, hyperglycemia, increase in fatty acid levels, hypertriglyceridemia, decrease in HDL-cholesterol, and increase in small dense LDL-cholesterol [6, 7]. In addition, the role of oxidative stress phenomenon independently or mediated by the metabolic disturbances in the progression of endothelial dysfunction is essential [8]. According to general agreed definition of metabolic syndrome as a cluster of these metabolic conditions, the relationship between development of endothelial dysfunction and metabolic syndrome has been recently strengthened and thus the presence of endothelial dysfunction has been commonly reported in these patients [9].

Recently, the use of different antioxidant agents such as vitamin E on improving endothelial cells functioning has been suggested which may be due to the effects of these supplements on reducing superoxide-dependent oxidative stress. Because of the near interaction between oxidative stress and the components of metabolic syndrome [10, 11], the beneficial effects of the consumption of vitamin E on repairing endothelial function via inhibiting oxidative stress in metabolic syndrome state is predictable. Furthermore, the cardiovascular and metabolic effects of omega-3 PUFAs are widespread which mediated by its preventive mechanisms including the decrease in serum level of triglyceride, as well as increasing in the level of HDL level [12, 13]. Also, as demonstrated in vitro, the use of omega-3 PUFAs has been accompanied with lower expression of adhesive molecules expression on endothelial cells, as well as releasing of endothelial relaxation properties [14]. In this regard, it seems that the use of these drug supplements may positively influence the endothelial function particularly in those who were affected by metabolic syndrome. Regarding the effects of these drug supplements on endothelial function, controversial results have been obtained especially in young people. Thus, the present study aims to explore the effects of vitamin E and omega-3 PUFAs on endothelial function indicators among adolescents with metabolic syndrome.

## 2. Methods

In a randomized, double blind, and placebo-controlled trial, we studied 90 young individuals, aged 10 to 18 years, who were referred to the pediatrics obesity clinic at the Isfahan Cardiovascular Research Center. All participants had the criteria of the metabolic syndrome, as described by the International Diabetes Federation (IDF) Definition for children and adolescents revised in 2007 as having the metabolic syndrome if they met three or more of the following criteria for age and sex: waist circumference (WC) > 90th percentile (or adult cut-off if lower) as assessed by waist circumference; triglycerides > 1.7 mmol/L or specific treatment for this lipid abnormality; HDL-cholesterol < 1.03 mmol/L or specific treatment for this lipid abnormality; systolic blood pressure (BP)> 130 or diastolic BP > 85 mm Hg or treatment of previously diagnosed hypertension; fasting blood glucose > 5.6 mmol/L (oral glucose tolerance test recommended) or known type 2 diabetes mellitus [15].

Those adolescents with any underlying genetic disorders, endocrine disorders, or any chronic disorders or who recently used drugs influencing body weight or took antioxidative medications were not included into the study. The investigation conformed to the principles outlined in the Declaration of Helsinki and also approved by the ethical committee of the Isfahan University of Medical Sciences. Written informed consent was obtained from all participants' parents.

The subjects were randomly assigned to receive either vitamin E tablets (400 IU/day, *n* = 30) or omega-3 tablets (2.4 gr/day, *n* = 30) or placebo (*n* = 30).

The placebo tablet was identical to the active supplement vitamin capsule. No physician, investigator, nurse, or patient could differentiate between the active treatment capsule and the placebo capsule. Taking pill counts, which revealed no essential irregularities, controlled the reliability of medication intake. All subjects maintained the same medication other than the study medication throughout the duration of the study. The duration of drugs administration was 8 weeks.

Before the beginning of drug regimens and also after its completion, the anthropometric parameters as well as serum levels of biochemical markers were assessed. The weight was measured using a single Seca digital weighing scale to the nearest 0.1 kg, and the height was measured using the Seca stadiometer, to the nearest 0.1 cm. Waist circumference was measured using a flexible tape measure at the natural indentation or at a level midway between the iliac crest and the lower edge of the rib cage if no natural indentation was visible. Waist circumference was recorded to the nearest 0.5 cm, and the mean of two measures within 1 cm of each other was used. Blood pressure was measured twice in the left arm by an examining physician using a mercury column sphygmomanometer (Korotkoff phases I and V) after the subject had been at rest in the seated position for 5 minutes. Fasting blood samples were also obtained at baseline and at the end of each drug regimen (Week 8). Plasma analysis for the lipid profile (using enzymatic test), fasting glucose (using glucose oxidase test), and insulin (using ELISA test) was done in laboratories of Isfahan Cardiovascular Research Institute. The samples were collected in Vacutainer tubes for serum separation and centrifuged at 3000 rpm for 15 minutes (4°C). The serum was collected and stored at −70°C until use.

For assessing endothelial functional state, the parameter of vascular endothelial growth factor (VEGF) was selected. The serum level of VEGF was measured by ELISA test (Randox LAB, UK).

The study endpoint was to compare the changes in the measured parameters following studied drug regimens across the three study groups.

*Statistical Analysis*. Results were presented as mean ± standard deviation (SD) for quantitative variables and were summarized by absolute frequencies and percentages for categorical variables. Continuous variables were compared using one-way analysis of variance (ANOVA) and/or nonparametric or Kruskal-Wallis test whenever the data did not appear to have normal distribution or when the assumption of equal variances was violated across the three groups. Categorical variables were, on the other hand, compared using Chi-square test or Fisher's exact test when more than 20% of cells with expected count of less than 5 were observed. The changes in the parameters following treatment schedules compared with the baseline were assessed by the paired *t*-test or Wilcoxon test. The difference between VEGF at the first and end of study was calculated. The correlation between the quantitative variables was examined by Pearson's correlation coefficient test. The multivariable linear regression modeling was applied to assess the changes in VEGF values following treatments. For the statistical analysis, the statistical software SPSS version 19.0 for windows (SPSS Inc., Chicago, IL) and the statistical package SAS version 9.1 for windows (SAS Institute Inc., Cary, NC, USA) were used. *P* values less than 0.05 were considered statistically significant.

## 3. Results

Regarding completion rate of treatment schedules, 30 patients in vitamin E group, 26 in omega-3 group, and 27 in placebo group completed their treatment course. No differences were found across the three study groups with regard to gender ratio, average age, and mean anthropometric parameters at baseline, as well as baseline levels of serum lipids and fasting blood sugar. However as clearly shown in Table 1, the use of omega-3 supplementation for eight weeks led to significant increase in serum HDL level compared with the group treated with vitamin E or placebo group (40.80 ± 7.63, 44.06 ± 5.94, and 37.17 ± 3.93, *P* = 0.007). The increase in body weight and height appeared homogenously in all three groups overtime. Although the baseline level of VEGF was significantly lower in both groups which were administered vitamin E or omega-3 compared with the placebo group, supporting statistical analyses revealed a stronger reduction of VEGF plasma values after administration of omega-3 (Table 2). Univariate analyses using Pearson's test revealed significant direct correlations between the change in VEGF value and only serum FBS level (Table 3). In this regard, no significant correlations were found between the change in VEGF and baseline levels of other markers including anthropometric indices and serum lipids. Using multivariable linear regression model (Table 4) and considering placebo group as the reference, the administration of omega-3 could significantly reduce VEGF with the presence of other baseline variables (Beta = −12.55, *P* = 0.012).

## 4. Discussion

Our results demonstrated that administration of omega-3 can effectively improve endothelial function in adolescents with metabolic syndrome by reducing the level of serum VEGF for atherosclerosis progression and endothelial destabilization. In the present study, we focused on endothelial dysfunction state among adolescents with metabolic syndrome because the different physiological and pathological aspects of metabolic syndrome and its related vascular mechanisms have been already questioned. In this context, changes in vascular endothelial function in these populations as well as its therapeutic approaches are now under investigation. Because of the central role of oxidative stress in development of endothelial dysfunction [3], employing antioxidant supplements to improve endothelial function in children and adolescents with metabolic syndrome can result in preventing cardiovascular disorders in the future. In this regard, we assessed the effects of two common drug supplements of vitamin E and omega-3 on endothelial dysfunction and found significant improvement in VEGF index as a major indicator of endothelial function, while the beneficial effect of vitamin E on endothelial function was not documented.

Long-chain omega-3 PUFAs is richly found in fatty fish and fish oil. The beneficial effects of these types of fatty acids on relaxation of vascular endothelium as well as promotion of vascular compliance have been widely investigated [16, 17]. Our study could support an early and consistent improvement in endothelium-dependent dilatation following treatment with the omega-3 PUFAs in young people with metabolic syndrome. Our findings are in line with a previous study that was done among patients with hypercholesterolemia [18]. The mechanism of this phenomenon is not fully understood, but it has been suggested that omega-3 PUFAs may protect vascular system against vasospasm and thrombosis by enhancing nitric oxide release and prostacyclin synthesis, and also by suppressing thromboxane formation [19].

Although some studies on human models with various cardiovascular risk factors showed that the use of omega-3 could improve endothelial function, the optimal dose and duration of treatment are still unclear. In the present study we showed that the use of omega-3 2.4 grams/day for a short period of 8 weeks could effectively improve endothelial function in metabolic syndrome condition. In some studies, choosing an oral dose of 0.8 g/kg/day of omega-3 supplement could be effective to point therapeutic effects of omega-3 on vascular function and thus preventing atherosclerosis [20]. A meta-analysis concluded that improving endothelial function may be achieved by applying a dose of omega-3 ranging from 0.45 to 4.5 g/d over a median of 56 days [21]. According to our observation, considering notably lower dosages of this supplement as 2.4 grams/day can be enough to unveil its effects on endothelial function in young population.

In our study, we selected VEGF index for assessment of endothelial function because it has been demonstrated that although VEGF-A can stimulate endothelial cell survival, endothelial migration and proliferation, generation of nitric oxide, mitosis of endothelial cells, the induction of angiogenesis, and vasodilatation [22], the level of VEGF has been associated with atherosclerosis progression and lesion destabilization. In metabolic syndrome state, endothelial dysfunction seems to be the substantial factor responsible for the vascular complications in subjects with metabolic syndrome, which manifests in increased plasma level of VEGF and decreased plasma level of nitric oxide [23]. In some population-based studies, a positive association was shown between trends in levels of plasma VEGF and metabolic syndrome and its components [24]. Therefore, the use of omega-3 can be proposed as a VEGF antagonism and a novel pharmacological approach for metabolic syndrome and its components, targeting the lipid-transport properties of the endothelium to improve muscle insulin sensitivity and glucose disposal [25].

Our study had some potential limitations, including consideration of a small sample size affecting power of the study especially power of the multivariable analysis, applying only IDF definitive criteria for metabolic syndrome and thus ignoring other definitions with the different ability to estimating and determining this syndrome, and also no programming different doses and treatment periods to achieve optimal effects of drug supplements on endothelial function.

In conclusion, the administration of omega-3 can effectively improve endothelial function in children and adolescents with metabolic syndrome assessed by measuring serum VEGF value; however, this effect was not confirmed following administration of vitamin E. Regarding effects of omega-3 on lipid profile, the major effect of this supplement is associated with elevating serum HDL. For determining the best therapeutic drug regimen, examining different dosages of the supplements with the different follow-up periods should be considered in future studies.

## Figures and Tables

**Table 1:** Anthropometric and biochemical parameters in the three study groups.

Variable	Vitamin E group (*n* = 30)	Omega-3 group (*n* = 26)	Placebo group (*n* = 27)	*P* value
Sex (men/women) %	46.2/53.8	66.7/33.3	74.1/25.9	0.841
Mean age (year)	16.15 ± 1.89	17.14 ± 2.82	16.63 ± 2.39	0.589
Weight (baseline), kg	65.82 ± 17.35	57.08 ± 14.22	67.02 ± 18.79	0.071
Weight (final), kg	67.28 ± 16.85	58.46 ± 13.63	70.14 ± 18.19	0.033
*P* value	0.069	<0.001	0.001	
Height (baseline), cm	153.40 ± 11.13	148.98 ± 10.24	156.15 ± 12.46	0.073
Height (final), cm	154.70 ± 11.24	151.85 ± 9.82	158.04 ± 12.03	0.073
*P* value	<0.001	<0.001	0.001	
Waist circumference (baseline), cm	93.87 ± 10.88	89.50 ± 8.77	95.44 ± 12.50	0.303
Waist circumference (final), cm	95.04 ± 11.23	89.54 ± 8.42	97.08 ± 11.67	0.341
*P* value	0.754	0.932	0.225	
Cholesterol (baseline), mg/dL	183.27 ± 47.46	178.68 ± 38.07	197.50 ± 33.41	0.248
Cholesterol (total), mg/dL	192.00 ± 44.46	195.11 ± 42.61	192.83 ± 32.60	0.978
*P* value	0.563	0.508	0.833	
Triglyceride (baseline), mg/dL	179.63 ± 99.19	162.36 ± 59.78	218.62 ± 111.87	0.111
Triglyceride (total), mg/dL	197.80 ± 63.00	165.87 ± 87.58	198.49 ± 49.98	0.279
*P* value	0.447	0.998	0.129	
HDL (baseline), mg/dL	38.00 ± 8.55	39.16 ± 10.67	33.21 ± 3.39	0.066
HDL (total), mg/dL	40.80 ± 7.63	44.06 ± 5.94	37.17 ± 3.93	0.007
*P* value	0.303	0.276	0.068	
LDL (baseline), mg/dL	103.96 ± 26.47	107.20 ± 28.27	119.83 ± 26.21	0.128
LDL (total), mg/dL	116.20 ± 42.46	123.22 ± 47.26	125.55 ± 31.82	0.870
*P* value	0.438	0.177	0.226	
Fasting blood sugar (baseline), mg/dL	90.40 ± 22.30	88.84 ± 8.05	81.30 ± 8.71	0.089
Fasting blood sugar (total), mg/dL	85.70 ± 11.27	99.37 ± 37.43	80.33 ± 4.42	0.146
*P* value	0.644	0.346	0.641	

**Table 2:** The baseline and final values of VEGF in the three study groups.

Parameter	Vitamin E group (*n* = 30)	Omega-3 group (*n* = 26)	Placebo group (*n* = 27)	*P* value
VEGF (baseline), pg/dL	249.93 ± 206.60	211.96 ± 128.34	133.67 ± 118.06	0.013
VEGF (final), pg/dL	227.05 ± 340.61	103.95 ± 77.77	102.25 ± 75.52	0.073
*P* value	0.308	0.001	0.679	
ΔVEGF, pg/dL	32.59 ± 151.04	132.79 ± 113.35	24.76 ± 105.99	

VEGF: vascular endothelial growth factor.

**Table 3:** Association between VEGF index and other baseline variables.

Parameter (baseline)	Correlation coefficient (*r*)	*P* value
Body weight	−0.170	0.276
Height	−0.163	0.297
Waist circumference	−0.114	0.468
Serum cholesterol	0.059	0.715
Serum triglyceride	0.065	0.686
Serum HDL	0.271	0.095
Serum LDL	−0.011	0.949
Serum FBS	0.321	0.044

**Table 4:** The multivariate linear model for assessment of the effect of omega-3 on VEGF.

Parameter (baseline)	Beta	Standard Error	*P* value
Omega-3 use	−125.470	45.168	0.012
Body weight	−10.303	7.725	0.197
Height	8.004	6.832	0.255
Waist circumference	10.646	6.844	0.135
Serum cholesterol	−3.522	0.2691	0.205
Serum triglyceride	0.939	0.518	0.085
Serum HDL	4.607	2.722	0.106
Serum LDL	2.131	1.215	0.225
Serum FBS	1.165	3.136	0.715
